# A western United States snow reanalysis dataset over the Landsat era from water years 1985 to 2021

**DOI:** 10.1038/s41597-022-01768-7

**Published:** 2022-11-07

**Authors:** Yiwen Fang, Yufei Liu, Steven A. Margulis

**Affiliations:** Department of Civil and Environmental Engineering, University of California, Los Angeles, Los Angeles, CA USA

**Keywords:** Cryospheric science, Water resources, Cryospheric science, Cryospheric science, Hydrology

## Abstract

Water stored in mountain snowpacks (i.e., snow water equivalent, SWE) represents an important but poorly characterized component of the terrestrial water cycle. The Western United States snow reanalysis (WUS–SR) dataset is novel in its combination of spatial resolution (~500 m), spatial extent (31°–49° N; 102°–125° W), and temporal continuity (daily over 1985–2021). WUS–SR is generated using a Bayesian framework with model-based snow estimates updated through the assimilation of cloud-free Landsat fractional snow-covered area observations. Over the WUS, the peak SWE verification with independent *in situ* measurements show correlation coefficient, mean difference (MD), and root mean squared difference (RMSD) of 0.77, −0.15 m, and 0.28 m, respectively. The effects of forest cover and Landsat image availability on peak SWE are assessed. WUS–SR peak SWE is well correlated (ranging from 0.75 to 0.91) against independent lidar-derived SWE taken near April 1^st^, with MD <0.15 m and RMSD <0.38 m. The dataset is useful for characterizing WUS mountain snow storage, and ultimately for improving snow-derived water resources management.

## Abstract (summary)

**Table:** 

Measurement(s)	snow water equivalent, snow depth, fractional snow covered area
Technology Type(s)	Bayesian snow reanalysis framework with assimilation of fractional snow covered area
Sample Characteristic - Location	Western United States

## Background & Summary

Water stored in seasonal snowpacks, typically expressed in the form of snow water equivalent (SWE), provides a key resource relevant to water supply, hydropower generation, agricultural irrigation, river navigation, and urban usage in many areas of the globe. In the Western U.S. (WUS) it is estimated that more than half of runoff comes from seasonal snowmelt^1,2^. Knowledge of SWE and its space-time variability impacts food, water and energy security, the financial stability of hydropower utilities, and public safety^3–5^.

*In situ* SWE data, even in the WUS where it is arguably most readily collected operationally, remains extremely sparse. Moreover, snow exhibits significant spatial heterogeneity due to variability in snowfall, redistribution and ablation controlled by local meteorological conditions, landcover, forest cover, and other physiographic characteristics^6^, especially in mountainous regions with high terrain complexity. The *in situ* snow stations that do exist are typically located in forest clearings, mid-elevations and flat terrain that do not necessarily sample the underlying heterogeneity of SWE^7,8^. Hence, *in situ* networks tend to provide an incomplete picture of the spatial patterns of SWE and how point-scale SWE integrates to basin-scale water volumes.

Remotely-sensed (satellite or airborne) observations of snow provide the potential to sample spatially-distributed characteristics of snow. The historically available satellite-borne measurements most closely related to SWE use Passive Microwave (PM, e.g., AMSR-E, SSMI) measurements to infer SWE or snow depth. However, PM measurements are typically obtained at coarse resolutions (tens of kilometers and thus incapable of resolving finer scale heterogeneity) and are highly sensitive to snowpack stratigraphy and microstructure, wet snow, and forest coverage (introducing significant uncertainty and bias into SWE estimates^9^). Recent and future airborne and spaceborne concepts aim to measure snow depth (from lidar^10,11^, photogrammetry, radar^12^), or SWE (from P-band^13^, C-band, X-band^14^, and Ku-band radar^15^). These newer methods show promise but cannot yet provide a long-term spatially-distributed SWE record.

To leverage remotely-sensed and *in situ* datasets relevant to snow processes, data assimilation combined with snow and land surface models (LSMs) can be used to constrain model estimates based on snow related observations. Global reanalysis products including ERA5^16^, ERA5-land^17^, JRA55^18^, GLDAS^19^, MERRA2^20^, and GlobSnow v3.0^21^ estimate terrestrial snow accumulation and melt with commonly used LSMs (e.g., VIC, SiB, Catchment, Noah) at scales of ~ 0.1° to 1°. Though coarse resolutions are typical in global applications, they do not provide the desired resolution to capture spatial variations, especially in complex terrain^22^. Additionally, several studies have found large uncertainties in SWE volumes derived from various input forcings and models applied over global snow covered mountains^23,24^. Snow-focused products over the U.S. using data assimilation include the Snow Data Assimilation System (SNODAS^25^) product and the University of Arizona SWE dataset (UA^26^). SNODAS daily SWE estimates are available from 2004 at the spatial resolution of 1 km × 1 km. UA daily SWE estimates start from 1982 at the spatial resolution of 4 km × 4 km. Hence, SNODAS has a more limited temporal coverage (less than 20 years), and UA is at relatively coarse resolution that can be suboptimal for assessing spatial variability in mountainous domains. In the mountainous WUS, historical space–time continuous snow estimates at high to moderate resolution and with low uncertainty are needed.

**Fig. 1:**
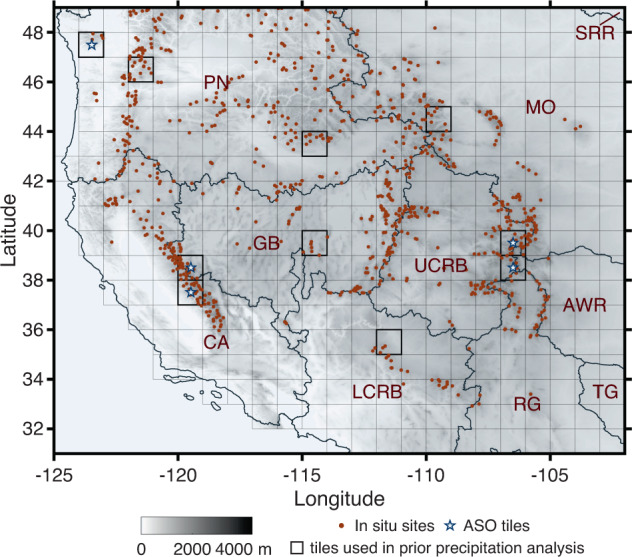
Map of elevation (meters) over the WUS domain with snow reanalysis tiles (1° × 1° squares) and Hydrologic Unit Codes 2 (HUC2) basins. HUC2 basins include California (CA), Pacific Northwest (PN), Great Basin (GB), Upper Colorado River Basin (UCRB), Missouri (MO), and other basins, i.e., Lower Colorado River Basin (LCRB), Rio Grande (RG), Texas Gulf (TG), Arkansas-White-Red (AWR), and Souris-Red-Rainy regions (SRR). The tiles highlighted in bold black outlines (in total 10) are used for prior precipitation uncertainty analysis as described in the *Methods* section. *In situ* SWE sites and tiles with ASO SWE estimates (used for verification) are illustrated with red dots and white stars, respectively.

To fill this gap, we use a Bayesian data assimilation approach that leverages high-resolution remotely-sensed visible and near infrared (Vis-NIR) measurements that provide information on fractional snow-covered area (fSCA) and how its seasonal evolution is related to SWE. Specifically, the approach yields a new snow reanalysis dataset across the WUS (Fig. 1) over the Landsat–era (water years (WYs) 1985 to 2021). The dataset is publicly available at National Snow and Ice Data Center (10.5067/PP7T2GBI52I2)^27^. The daily snow reanalysis framework accounts for a priori uncertainties in meteorological forcings and other snow model parameters and reduces the uncertainty via a Bayesian data assimilation approach as described in more detail in the Methods section. The snow reanalysis SWE estimates are verified against independent *in situ* SWE measurements and lidar-based SWE products. Previous applications of the method over the Sierra Nevada have demonstrated the ability to characterize historical snow droughts, characterize snowfall estimates from SWE accumulation patterns, and improve streamflow predictions^28–31^.

## Methods

### Snow reanalysis framework

**Fig. 2:**
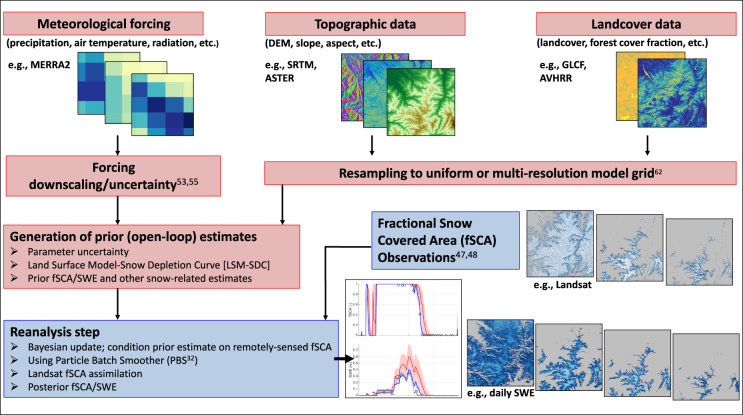
Flowchart for the Bayesian snow reanalysis framework used to generate the WUS–SR dataset (adapted from Margulis *et al*.^34^).

A Bayesian “snow reanalysis” framework^32–34^ (Fig. 2) is applied to generate a new Landsat-era dataset over the WUS, herein referred to as the Western U.S. – Snow Reanalysis (WUS–SR). The dataset contains space–time continuous SWE and fractional snow-covered area (fSCA) estimates constrained by remotely-sensed (Landsat) fSCA using a particle batch smoother (PBS) data assimilation technique.

The snow reanalysis framework generates an ensemble of (equally likely) prior snow estimates using a land surface model (LSM) driven by meteorological forcing, topographic data and landcover data (red boxes in Fig. 2). Uncertainty is expressed via perturbations related to precipitation (snowfall), the snow depletion curve and snow albedo in each ensemble member (described in the Uncertainty Parameters and Measurement Error section). The reanalysis step assimilates Landsat-derived fSCA measurements to provide posterior snow estimates (blue boxes in Fig. 2). More specifically, the a priori (equal) weights are updated to posterior weights that reflect the likelihood that a given ensemble member fits the fSCA measurements^34^. The posterior weights, when combined with the prior ensemble estimates (e.g., SWE) can be used to derive posterior estimates (ensemble statistics) of the relevant states. The resulting posterior SWE, snow depth and fSCA make up the published dataset.

While the snow reanalysis framework could be applied with any LSMs and their static and dynamic model inputs, in creating the WUS–SR, we use the same setup as described in Margulis *et al*.^34^. In summary, a spatially-distributed version of the SSiB–SAST LSM^35–37^ using the BATS^38^ snow albedo model and coupled with the Liston^39^ Snow Depletion Curve (SDC) model is used. The SSiB–SAST LSM models a three-layer snowpack when snow depth is above 5 cm, and uses a one-layer scheme when snow depth is below 5 cm. SWE at each layer is computed by mass balance with components including snowfall, rainfall, snowmelt, runoff and evaporation at the snow surface layer^36^. Snow density, and therefore snow depth, is determined by the SAST compaction process as described in Sun and Xue^35^. For computational reasons, a uniform spatial resolution of 16 arcseconds (~500 m) is chosen with hourly outputs aggregated to the daily time step using an ensemble of 50 members. The SDC provides the mechanism whereby modeled estimates of SWE (and its sub-grid heterogeneity) provide predicted estimates of fSCA. For the reanalysis, the LSM–SDC model is applied separately at each pixel to the bare snow and forest covered fractions. It is assumed that Landsat sensors cannot see snow under the forest canopy. Therefore, only the predicted fSCA over bare soil is compared to the Landsat fSCA measurements in the assimilation step for each grid, while the update is applied to both bare and forested pixel fractions to obtain the grid-averaged SWE^34^. The Bayesian update is applied in a batch over one WY at a time, where the batch of fSCA measurements from that year are used together to derive the posterior weights and estimates. Table 1 summarizes the models and method used in the snow reanalysis framework.

**Table 1:** Modules used in the snow reanalysis framework.

	Model/Methods	Notes
Prior step	SSiB-SAST	Land Surface Model (LSM) framework; surface (snow) energy balance fluxes; BATS snow albedo module
	Liston Snow Depletion Curve (SDC)	Sub-grid distribution and pixel averaged SWE, ablation, and fSCA
Posterior step	Data assimilation (PBS)	Ensemble-based Bayesian updated via assimilation of Landsat fSCA measurements

### Land surface model inputs

To generate the WUS–SR dataset, globally-available datasets are used as inputs. This includes the MERRA2^20^ near-surface meteorological forcing data, 30-m Shuttle Radar Topography Mission (SRTM^40,41^) digital elevation model (DEM) for topographic data (with gaps filled by the Advanced Spaceborne Thermal Emission and Reflection, ASTER^42^, version 2), 1-km Advanced Very High Resolution Radiometer (AVHRR^43,44^) landcover data and 30-m Global Land Cover Facility (recently updated to the Landsat Tree Canopy Version 4, TCC^45,46^) forest cover fraction data. The TCC data is available in 2000, 2005, 2010, and 2015 where each timestamped year represents multi-year average forest cover conditions during that period. Rather than implementing time-varying forest cover, the time-averaged forest cover over these 4 composites is applied for the whole reanalysis period. All inputs are downscaled or aggregated to the chosen model resolution. For example, the 1-km AVHRR dataset is first interpolated to the 30-m resolution of the raw SRTM DEM at the nearest grid and then aggregated to the 480-m model resolution.

The meteorological forcings used in this dataset include 2-m air temperature, 2-m specific humidity, 10-m zonal and meridional wind speed, surface pressure, surface precipitation, and surface downwelling shortwave^34^. The raw MERRA2 precipitation is perturbed to account for the expected bias and uncertainty in snowfall inputs (see more detail in the Uncertainty Parameters and Measurement Error section). In addition to precipitation, the bias and uncertainties of MERRA2 air temperature, dew point temperature (computed from MERRA2 specific humidity), and shortwave radiation are represented via ensemble perturbations. Hourly snowfall is computed by downscaled and bias-corrected air temperature and precipitation using a rain–snow threshold of 2 °C. When air temperature is below the threshold, precipitation is classified as snowfall. Table 2 summarizes the static and dynamic inputs used to generate the dataset, as well as assimilated data described in the next section.

**Table 2:** Static and dynamic model inputs and assimilated data.

Inputs	Dataset	Resolution
Static Inputs	SRTM^40,41^	1 arcsecond × 1 arcsecond
	ASTER DEM^42^	1 arcsecond × 1 arcsecond
	AVHRR landcover^43,44^	1 km × 1 km
	GLCF forest cover^45,46^	30 m × 30 m
Meteorological Forcing	MERRA2^20,63^	0.5° × 0.625°
Assimilated Data	Landsat Imagery^64^	30 m × 30 m

### Assimilated landsat fSCA data

The timeseries of derived Landsat fSCA (raw resolution of ~30 m aggregated to 16 arcseconds), over the course of a WY is used as the measurement constraint in the Bayesian particle batch smoother (PBS) update. The retrieval of Landsat fSCA is obtained through applying linear spectral unmixing methods described in Painter *et al*.^47^ and Cortés *et al*.^48^ using Vis-NIR reflectance measured from three Landsat satellites: 1) Landsat 5 Thematic Mapper (TM) from 1985 to 2011; 2) Landsat 7 enhanced Thematic Mapper (ETM+) from 1999 to present, and 3) Landsat 8 Operational Land Images (OLI) from 2013 to present. Orbital characteristics of the Landsat platform provide swath images every 16 days (~23 images per year). Adjacent swaths contain some overlap such that some locations may have up to ~46 fSCA images from a single satellite per year. This is the typical number of available measurements from WYs 1985–1999 (when only Landsat 5 is available) and in 2012 (when only Landsat 7 is available). In the other years, where two satellites are available (i.e., WYs 2000–2011 and 2013–present), the number of available measurements is doubled. The failure of Landsat 7 Scan Line Corrector (starting in 2003) removes ~22% of its image areas, thus reducing the number of measurements per year (USGS^49^). However, the number of measurements described above provide only an upper limit on those used in the WUS–SR. Cloud contamination can significantly reduce the number of available (assimilated) measurements. Following the cloud screening methods described in Margulis *et al*.^34^ and Liu *et al*.^50^, the internal Landsat cloud mask is used to attempt to exclude images with cloud cover fraction greater than 40%. For those images included, the internal cloud masks are used to screen out any cloudy pixels. Thus, areas identified as contaminated by clouds are removed before assimilation which reduces the available number of measurements. Though errors introduced by omission or commission are inevitable, they are implicitly accounted for in the snow reanalysis framework as described in the *Measurement Error* section below.

**Fig. 3:**
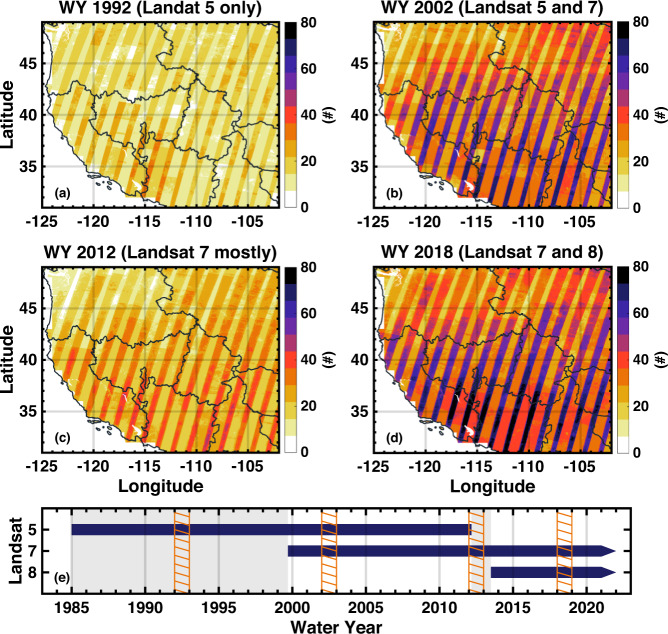
Illustration of the number of cloud-free Landsat measurements used in the WUS–SR for four WYs (top four panels) and Landsat mission timelines (bottom panel). Landsat images with diagnosed cloud fractions of 40% are excluded entirely and those will less than 40% use the Landsat cloud mask to screen out cloudy measurements. The four illustrative WYs include: (**a**) WY 1992 and (**c**) 2012 when one Landsat satellite is in orbit, and (**b**) WY 2002 and (**d**) 2018 when two Landsat satellites are in orbit. The stripes showing a larger number of measurements are the overlapping areas between adjacent Landsat tiles. The temporal coverage of measurements in (**e**) shows the Landsat 5, 7 and 8 mission timelines. Periods with only one Landsat satellite are shaded with a gray background. The orange hatched areas indicate the four WYs present in maps in the top panels.

Figure 3 shows that the total numbers of cloud-free fSCA measurements are much fewer in WYs 1992 and 2012 when only one Landsat platform is available over 10 months of the WY compared to WYs 2002 and 2018 when two Landsat satellites are available over the full WY. The number of available fSCA measurements is associated with satellite swaths that may cause spatial artifacts in posterior estimates within a WY. Grid cells with no fSCA measurements (no assimilation) or limited fSCA measurements may yield inconsistent results with grid cells that have abundant fSCA measurements. In the PN, spatial artifacts in the SWE estimates are more frequently observed when only one Landsat is available, where cloudy days are more common in the melting season. The method is capable of jointly assimilating other fSCA data (e.g., MODIS^34^) or other relevant snow data (e.g., snow depth^51^). The dataset presented herein is chosen to use Landsat-only data to provide a long-term homogeneous snow reanalysis product.

### Uncertainty parameters and measurement error

The ensemble Bayesian framework described above is applied by considering and modeling key sources of uncertainty and error. These include uncertainty in meteorological inputs and model parameters controlling snow albedo and sub-grid distribution, and fSCA measurement errors as described in more detail below and which follow those in Margulis *et al*.^34^.

#### Perturbed meteorological forcings

The a priori meteorological forcing uncertainties are embedded in the prior ensemble via perturbations to the nominal (MERRA2) inputs using parameters randomly generated from specified distributions. The uncertainty and bias correction models used are similar to those that have been successfully applied to the Sierra Nevada^33^, Andes^52^, and High Mountain Asia^50^ to downscale and perturb forcings. Forcing downscaling uses a topographic correction approach following Girotto *et al*.^53^. The raw MERRA2 forcings are first (bilinearly) interpolated to the snow reanalysis grid followed by an elevation correction using differences between the (coarser resolution) MERRA2 and (higher resolution) reanalysis DEMs. Downscaled precipitation, air temperature, dew point temperature and shortwave inputs are bias-corrected and perturbed using the formulation^34^:

1 $${PPT}_{j}={b}_{j}\cdot {PPT}_{MERRA{\rm{2}}}$$

2 $${T}_{a,j}={T}_{a{\rm{,}}MERRA{\rm{2}}}{\rm{+}}{\varepsilon }_{{T}_{a,j}}$$

3 $${T}_{d,j}={T}_{d{\rm{,}}MERRA{\rm{2}}}+{\varepsilon }_{{T}_{d,j}}$$

4 $${SW}_{j}=\left({\rm{1}}-{\gamma }_{j}\right)\cdot {SW}_{MERRA{\rm{2}}}$$

where *PPT*, *T*_*a*_, *T*_*d*_, and *SW* represent the precipitation, air temperature, dew point temperature, and shortwave radiation forcings respectively, the subscript *j* represents the perturbed forcing realization within the ensemble and *MERRA2* represents the downscaled MERRA2 forcings using the downscaling described above. The random variable *b* represents a lognormally distributed multiplicative precipitation perturbation. The random variable *ε*_*Ta*_ and *ε*_*Td*_ represent normally distributed additive error perturbations of air temperature and dew point temperature, respectively. The random variable *γ* represents a normally distributed multiplicative shortwave perturbation that varies with solar index (*SI*, ratio of MERRA2 solar radiation over clear sky solar radiation) to account for varying errors under clear-sky vs. cloudy-sky conditions^34^.

The moments of the precipitation parameter *b* distribution are estimated based on the same methodology described in Liu *et al*.^54^ from a sub-sample of 10 tiles across the WUS spanning a range of physiography and climatology (Fig. 1 in bold boxes). The precipitation uncertainty is quantified by running the snow reanalysis framework using a uniform (i.e., “uninformative”) distribution for the parameter *b*~*U*(0.1, 5) at the 10 tiles. After assimilating fSCA measurements using the PBS approach, a log-normal distribution is fitted to the posterior *b* values from all pixels and replicates in those 10 tiles (Table 3). The fitted distribution is then treated as the prior distribution for the full WUS–SR domain.

**Table 3:** Distributions of meteorological forcings and model parameter perturbations with details described in the Uncertainty Parameters and Measurement Error section.

Parameters	Distribution	Uncertainty Parameter Distributions Statistics		
**Meteorological Forcings**				
*b* (PPT)	Log normal	**Mean**	**CV**	
		1.80	0.69	
*ε*_*Ta*_ (T_a_)	Normal	**Mean**	**Std. Dev**.	
		0.85 K	1.39 K	
*ε*_*Td*_ (T_d_)	Normal	**Mean**	**Std. Dev**.	
		−1.37 K	1.20 K	
*γ* (SW)	Normal	**Mean**	**Std. Dev**.	**SI**
		0.2548	0.39	<0.5
		3.66 × SI3 - 1.88 × SI2 + 1.39 × SI - 0.050	−0.39 × SI + 0.58	0.5 to 1
		0	0.19	>1
**Model Parameters**				
*C*_*vis*_ (*α*_*vis*_)	Uniform	**Minimum**	**Maximum**	
		0.2	0.45	
*β* (SDC)	Uniform	**Minimum**	**Maximum**	
		0.05	0.8	

Std. Dev. Represents standard deviation and CV represents the coefficient of variation.

The derivation of uncertainty models for air temperature, dew point temperature and shortwave uncertainty analysis followed Girotto *et al*.^53^ by comparing downscaled MERRA2 forcings to *in situ* measurements across the WUS. The uncertainties of MERRA2 forcings are quantified based on *in situ* Snow Telemetry network (SNOTEL) and Soil Climate Analysis Network (SCAN) air temperature, shortwave, and dew point temperature measurements. For air temperature and dew point temperature, the differences between downscaled MERRA2 and *in situ* data (i.e., distribution of temperature errors *ε*_*Ta*_ and *ε*_*Td*_) are fitted with normal distributions separately. The *in situ* solar radiation measurements and downscaled MERRA2 data are used to fit normal distributions (Table 3) to the multiplicative parameter *γ* whose mean and standard deviation are polynomial functions of the *SI*.

Table 3 summarizes the fitted parameters of the uncertainty models. The multiplicative precipitation factor *b* follows a lognormal distribution with mean of 1.80 and coefficient of variation (CV) of 0.69, which corrects the underestimation in raw MERRA2 precipitation used as input to the LSM. The normally distributed air temperature error *ε*_*Ta*_, has a positive mean of 0.85 K, while the dew point temperature error *ε*_*Td*_ has mean of −1.37 K. The quantified mean parameters identify (and correct) a cold and dry bias in the MERRA2 data before running the LSM-SDC to generate prior snow estimates.

#### Perturbed model parameters

The snow reanalysis framework additionally acknowledges sub-grid snow heterogeneity (resulting in fractional snow-covered area) and the uncertainties in snow albedo that result from different dust conditions.

The sub-grid distribution of snow cover and SWE is captured by the Liston^39^ SDC model with the free parameter representing the coefficient of variation (*β*) of the lognormal distribution. The free parameter *β* itself is treated as a uniformly distributed (~*U*(0.05, 0.8)^34^) uncertainty parameter.

The uncertainties in snow albedo in the visible band are accounted for in the BATS snow albedo (*α*_*vis*_) model:

5 $${\alpha }_{vis,j}=\left({\rm{1}}-{C}_{vis{\rm{,}}j}{f}_{age}\right)\cdot {\alpha }_{VO}$$

where *C*_*vis*_ is a uniformly distributed (~*U*(0.2, 0.45)^34^) uncertainty parameter chosen to span clean to dusty snow conditions (Table 3). The variable *f*_*age*_ represents the fraction of snow albedo reduction due to snow aging. The fresh snow albedo *α*_*VO*_ is set to 0.95. Such an approach does not include any explicit information on dust, but instead tries to realistically span the uncertainty when dust may be present.

#### Measurement error

The data assimilation framework requires specification of fSCA error standard deviation as an input. The measurement error of retrieved Landsat fSCA is specified as 10% at ~500 m, which is consistent with previous work^34,50^. The measurement errors between different fSCA measurements are assumed to be uncorrelated in space and time.

## Data Records

The raw gridded 16 arcsecond (~ 500 m) daily snow reanalysis dataset over the WUS (WUS–SR) is publicly available at the National Snow and Ice Data Center (10.5067/PP7T2GBI52I2)^27^ in netCDF format. It starts from WY 1985 (Oct. 1^st^, 1984) to WY 2021 (Sept. 30^th^, 2021) and will be extended for future WYs when available (Table 4). The output files store daily maps of posterior SWE, fSCA, and snow depth within a 1° by 1° tile (Fig. 1) for a given WY. The results presented in this paper show the ensemble median of SWE (an output that is determined from the discrete PDF of posterior weights). The ensemble mean, standard deviation, and interquartile range of outputs are also provided in the dataset. Ancillary or derived data products (e.g., non-seasonal snow mask) are available upon request.

**Table 4:** Spatial and temporal information of the WUS–SR dataset.

Spatial Information	
Temporal Information	
Coverage	Northernmost: 49° N; Southernmost: 31° N
	Easternmost: 102° W; Westernmost: 125° W
Resolution	16 arcseconds (~ 500 m)
Distribution tile dimension	1° by 1°
Geographic coordinate system	WGS 84
Coverage	Oct. 1^st^, 1984–Sep. 30^th^, 2021 (i.e., WYs 1985–2021)
Output Resolution	Daily

## Technical Verification

**Fig. 4:**
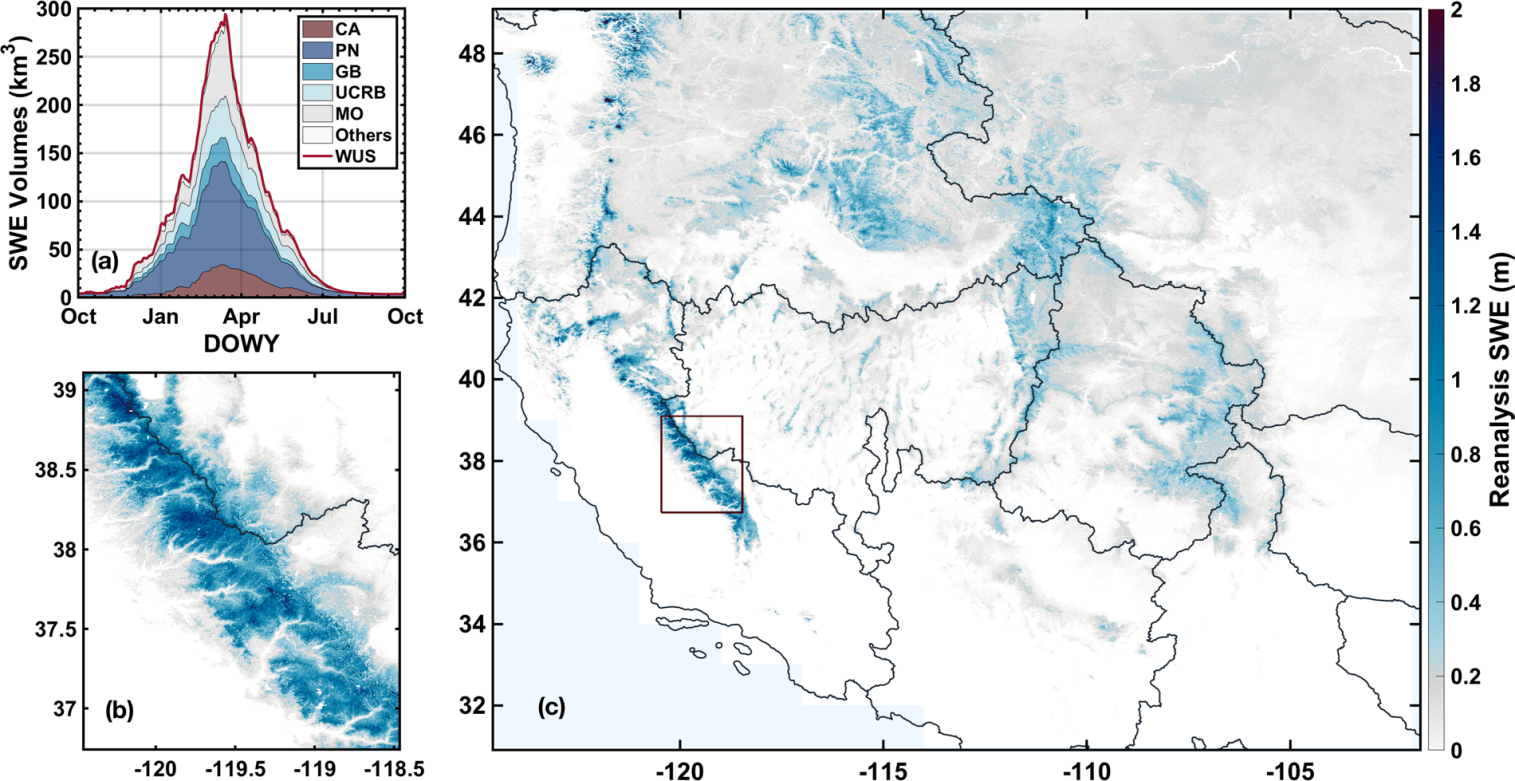
Illustrative results from the WUS–SR SWE estimates in WY 2019. (**a**) Seasonal cycle of SWE volume (km^3^) integrated over HUC2 basins. (**b**) Spatial distribution of SWE (meters) over part of the Sierra Nevada on March 1^st^, WY 2019. (**c**) Spatial distribution of WUS SWE (meters) on March 1^st^, 2019. The boxed area in (**c**) represents that shown in (**b**).

Figure 4 shows a sample of the seasonal cycle and spatial distribution of SWE over HUC2 basins and the entire WUS domain in WY 2019. No SWE or snow depth measurements are assimilated in deriving the WUS–SR dataset. Thus, *in situ* SWE and snow depth measurements, and ASO SWE and snow depth estimates are used as independent verification datasets. Landsat fSCA measurements are assimilated into the snow reanalysis framework assuming a measurement error (standard deviation) of 10%^34^. Though Landsat fSCA cannot be used for independent verification, the WUS–SR posterior fSCA estimates, which are fitted to these measurements using a likelihood function, are expected to have comparable bulk error. The snow reanalysis framework has been successfully applied previously to generate datasets over the Sierra Nevada, Andes, and High Mountain Asia^33,50,52^.

### Verification with *in situ* data

In this section, grid-averaged reanalysis SWE and snow depth are compared with point-scale *in situ* measurements. It should be acknowledged a priori that there are inevitable representativeness issues in the comparison between point-scale *in situ* data and grid-averaged snow reanalysis data. The WUS–SR estimates are modeled with assumed sub-grid heterogeneity within each ~500 m grid cell (which is modeled via a lognormal distribution) meant to account for the complex sub-grid variations in terrain (elevation, slope, aspect), forest cover, and meteorological forcings. Given that *in situ* stations are often sited in non-representative regions of a grid cell (i.e., in sheltered flat forest clearings), it is unlikely that the grid-averaged SWE/snow depth (spanning ~ 250,000 m^2^) should match the point-scale *in situ* SWE/snow depth (spanning ~10 m^2^). Nevertheless, *in situ* measurements, from the SNOTEL and CA Department of Water Resources (CADWR) networks, represent the best available data that covers much of the WUS and extends back several decades. While not expected to match each other, the verification herein is meant to illustrate consistency between the *in situ* measurements and WUS–SR estimates.

#### Peak SWE comparison with in situ data

*In situ* SWE measurements from WY 1985 to 2021 are taken from 1) the SNOTEL network (https://www.wcc.nrcs.usda.gov/snow/) managed by the U.S. Natural Resources Conservation Service (NRCS), and 2) CADWR (https://cdec.water.ca.gov/dynamicapp/staSearch from sensor type: “SNO ADJ (82)”), collections of automated snow pillows in the WUS. For *in situ* verification, we pair each *in situ* site with the closest snow reanalysis grid based on the geolocation of these two datasets. The precision of *in situ* coordinate values varies from 0.000001° (<1 m) to 0.01° (>1 km). Considering the potential for geolocation mismatch, the nine nearest pixels^32,33,55^ are additionally used to compare *in situ* and WUS–SR peak SWE. In this latter approach, the differences between *in situ* peak SWE and the neighboring WUS–SR grid cell peak SWE with the smallest difference among the nine nearest snow reanalysis grids are used. To compare the SWE on the same day, peak SWE day determined by *in situ* SWE is used to extract peak SWE from both datasets throughout the paper.

**Fig. 5:**
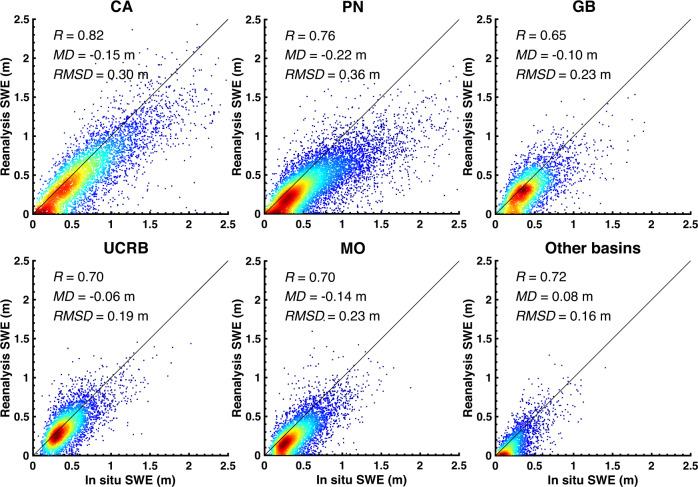
Density scatter plot of *in situ* (snow pillow) peak SWE and collocated posterior (grid-average) peak SWE grouped by HUC2 basins over WYs 1985 to 2021. The solid black line is the 1:1 line. The correlation coefficient (*R*), mean difference (*MD*), and root mean square difference (*RMSD*) are shown for each HUC2 basin. *In situ* data with peak SWE values greater than 1 cm are included in the comparison.

Figure 5 presents the density scatter plots comparing *in situ* peak SWE values against collocated grid-cell posterior peak SWE values. Peak SWE values less than 1 cm are screened out from the comparison. In total, 928 *in situ* sites are used in the comparison with the WUS–SR SWE estimates. To understand the performance of the WUS–SR dataset across different regimes in the WUS, verification is conducted for each HUC2 basin. The comparison is quantified using correlation coefficient (*R*), mean difference (*MD*), and root mean square difference (*RMSD*). Table 5 summarizes the number of total site-years, and statistics for both prior and posterior reanalysis SWE against *in situ* SWE within each HUC2 basin and over the WUS.

**Table 5:** Number of *in situ* sites and comparison metrics between *in situ* (snow pillow) peak SWE and collocated grid-averaged snow reanalysis prior and posterior (post.) peak SWE grouped by HUC2 basins.

HUC2	# Sites	# Site-years	*R*		*MD* (m)		*RMSD* (m)	
			Prior	Post.	Prior	Post.	Prior	Post.
CA	183	4911	0.75	0.82	−0.19	−0.15	0.36	0.30
PN	280	8566	0.76	0.76	−0.11	−0.22	0.31	0.36
GB	114	2776	0.49	0.65	−0.25	−0.10	0.34	0.23
UCRB	134	3823	0.51	0.70	−0.16	−0.06	0.26	0.19
MO	139	4114	0.47	0.70	−0.15	−0.14	0.27	0.23
Others	78	1736	0.59	0.72	0.19	0.08	0.25	0.16
WUS Total	928	25926	0.74	0.77	−0.16	−0.15	0.31	0.28

Comparison statistics including correlation coefficient (*R*), mean difference (*MD*), and root mean square difference (*RMSD*). *MD* is computed by subtracting snow reanalysis SWE from *in situ* SWE. A negative *MD* represents that the snow reanalysis peak SWE is less than mean of *in situ* peak SWE.

Compared with the performance of the prior peak SWE estimates (i.e., not constrained by Landsat fSCA), posterior SWE estimates show a better correlation (higher *R*) with less bias and random error (lower *MD* and *RMSD*) than the prior SWE over most of the HUC2 basins. Posterior SWE in CA has the highest correlation against *in situ* SWE (*R = *0.82). The correlations with *in situ* SWE over the entire WUS are improved from 0.74 (prior) to 0.77 (posterior). Posterior peak SWE in UCRB has lower bias and uncertainty compared against *in situ* data with a relatively small *MD* of 0.06 m in absolute value (reduced by 62% from prior *MD*) and *RMSD* of 0.19 m (reduced by 27%). Over the WUS, *in situ* peak SWE is (on average) larger than the WUS–SR peak SWE (negative *MD*). Sub-grid topographic variability, snow-forest interactions, and wind-driven snow redistribution may all cause differences seen between grid-averaged peak SWE and point-scale *in situ* peak SWE. The statistics for PN indicate comparable correlation of *in situ* and both prior and posterior snow reanalysis, however the *MD* and *RMSD* do not get improved from posterior to prior. Fewer cloud-free fSCA measurements are available in PN, which limits the improvement of snow reanalysis SWE via data assimilation.

**Fig. 6:**
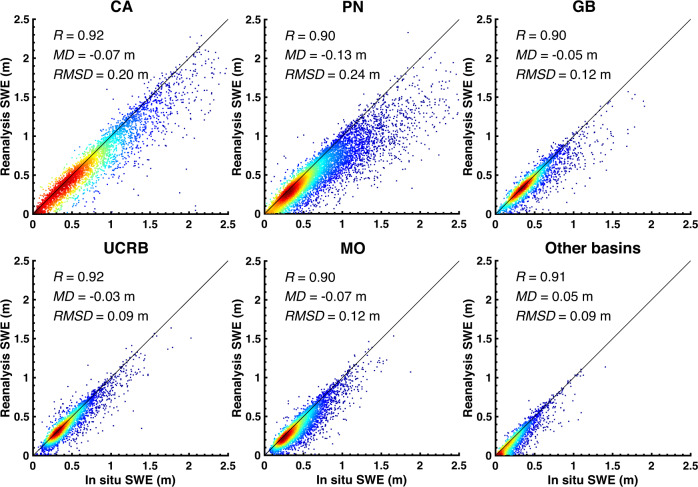
Same as the density scatter plot in Fig. 5 but using posterior (grid-average) peak SWE from the best match among nine closest neighbor pixels.

To acknowledge the potential geolocation mismatch, Fig. 6 provides verification of *in situ* peak SWE and posterior reanalysis peak SWE using an approach comparing to the best match among the nine nearest pixels. The WUS-wide correlation coefficient (*R*), *MD* and *RMSD* of posterior peak SWE and *in situ* peak SWE is 0.91, −0.08 m, 0.18 m, respectively. Compared to the approach used in Fig. 5, the posterior reanalysis peak SWE in Fig. 6 (as expected) is more correlated with *in situ* peak SWE (*R* values above 0.9), and has lower *MD* (<0.13 m) and *RMSD* (<0.24 m) over the WUS and at all HUC2 basins. Posterior reanalysis peak SWE is still lower than the *in situ* peak SWE at most of the sites, with the largest *MD* found in the PN. The PN has fewer cloud-free fSCA measurements, which may lead to larger errors than in regions with fewer cloud-contaminated images. The *MD* in CA is −0.07 m, which is within the range of −0.12 to 0.01 m as reported in Margulis *et al*.^33^, where the original 90-m Sierra Nevada SWE reanalysis was compared against *in situ* peak SWE using the same approach.

**Fig. 7:**
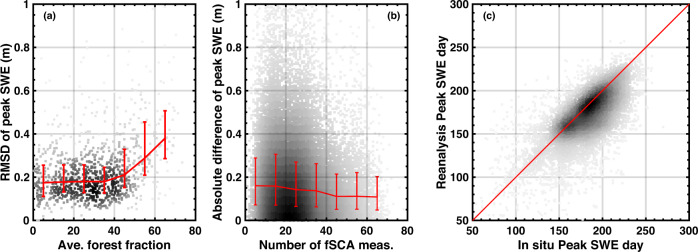
(**a**) *RMSD* of peak SWE as a function of averaged forest fraction for each site. *RMSD* is determined at each site from the 37-year peak SWE from *in situ* and posterior WUS–SR. (**b**) Absolute difference of peak SWE over the number of fSCA measurements (after cloud screening) for each year and site. The absolute difference of peak SWE is computed using *in situ* and posterior peak SWE. (**c**) Density scatterplot of peak SWE day from *in situ* and posterior WUS–SR for each year and site.

Figure 7a shows that the differences between posterior peak SWE and *in situ* peak SWE are sensitive to forest fraction exceeding 40%. The median *RMSD* remains stable at ~ 0.18 m for forest fractions below 40%, and gradually increases to ~ 0.38 m when forest fraction increases to over 60%. The larger *RMSD* at higher forest fraction pixels might be caused by 1) larger disparities between *in situ* sites (that tend to be in forest clearings) and collocated pixels with large averaged forest coverage fraction and/or 2) larger estimation errors in WUS–SR peak SWE in areas with large forest coverage. Aside from forest coverage effects, the difference between *in situ* and posterior peak SWE is impacted by the number of fSCA measurements as illustrated in Fig. 7b. When over 40 fSCA measurements (after cloud screening) are available, the median of absolute difference is as low as ~ 0.11 m. As the number of annual fSCA measurements is reduced, the median and spread of the absolute difference of peak SWE for each year increased. Figure 7c show that the peak SWE days determined by *in situ* data is highly correlated to peak SWE days determined by posterior WUS–SR SWE (*R* = 0.73). Overall, *in situ* SWE peaks later than the WUS–SR SWE with a *MD* value of −10 days.

#### Temporal (daily) SWE comparison with in situ data

**Fig. 8:**
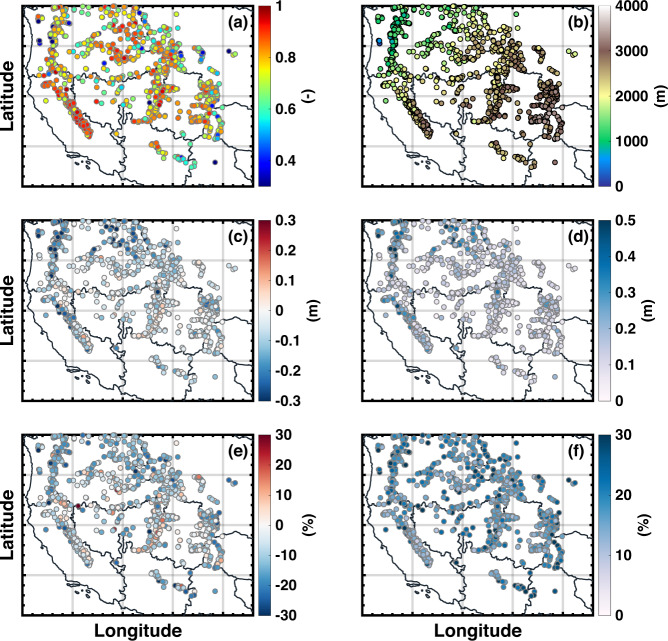
Spatial distribution of evaluation statistics determined via comparison of *in situ* daily SWE and collocated posterior SWE over WYs 1985 to 2021. Statistics include (**a**) *R*, (**c**) *MD* in meters, (**d**) *RMSD* in meters, (**e**) *MD* as percentage of peak SWE, and (**f**) *RMSD* as percentage of peak SWE. For reference, the *in situ* site elevations in meters are shown in (**b**). Daily SWE values less than 2.54 mm are excluded.

Figure 8 shows the spatial distribution of verification statistics at *in situ* sites by comparing posterior daily SWE against *in situ* daily SWE greater than 2.54 mm.

Over the entire WUS, posterior daily SWE at *in situ* sites have high correlations (median of 0.79), small *MD* (median of −0.08 m) and *RMSD* (median of 0.17 m) against *in situ* SWE. The comparison suggests that posterior daily SWE agrees reasonably well with daily *in situ* SWE, especially in CA and UCRB with higher correlations and relatively lower *MD* and *RMSD*. Daily posterior SWE is slightly lower than point-scale *in situ* SWE (Fig. 6b. negative *MD* in blue) at most of the sites. At some *in situ* sites in the western PN, posterior SWE shows higher differences. Figure 8(e,f) show that low *MD* and *RMSD* expressed as percent of peak SWE are observed at some sites with high *MD* and *RMSD* due to deep snow. For sites with both large absolute and percent of differences, some of these differences may represent larger errors caused by fewer available fSCA measurements after clouds screening. Finer resolutions may be needed to capture large sub-grid SWE values.

#### Peak snow depth comparison with in situ data

**Fig. 9:**
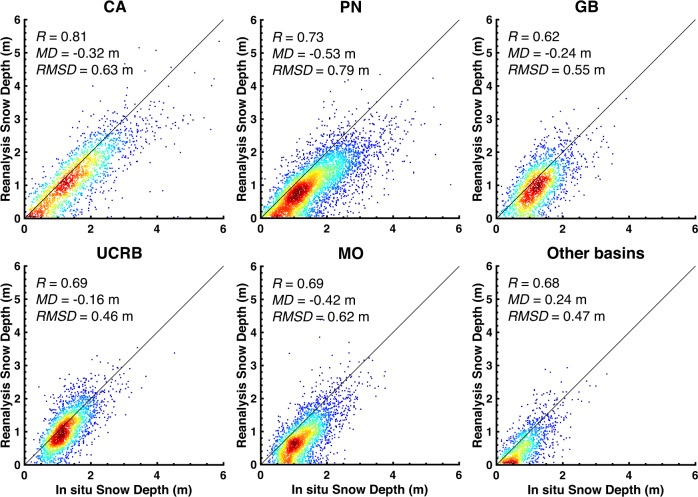
Same as Fig. 5 but for peak snow depth. Peak day is determined by *in situ* peak SWE. *In situ* data with peak snow depth values greater than 5 cm are included in the comparison.

*In situ* snow depth measurements are taken from the same sources as SWE (i.e., NRCS and CADWR from sensor type: “SNOW DP (18)”). Similar verification steps as with peak SWE (Fig. 5) are conducted for snow depth as shown in Fig. 9. Compared to the SWE measurements, however, *in situ* snow depth measurements appear to be of lower quality with some station-years showing snow depth with persistently high values throughout the year, non-physical oscillations in the measurements, and other erroneous behavior that are clearly inconsistent with the corresponding SWE measurements. Hence, extra screening is applied to the data before being used for verification. *In situ* snow depth measurements that changed by more than 1 m in a single day were assumed erroneous and excluded from the analysis. Further, assuming snow density is within the range of 200 to 500 kg/m^3^ at the peak day, snow depth measurements outside 2–5 times the corresponding SWE measurements were removed. To avoid incorrectly diagnosing peak snow depth day from snow depth measurements with missing data after screening, the *in situ* peak SWE day was used to determine the *in situ* snow depth used for comparison with posterior reanalysis estimates. Overall, posterior peak snow depth is correlated with *in situ* peak snow depth (*R* = 0.72) and has an *MD* of −0.36 m and *RMSD* of 0.66 m over the WUS. Compared to the results from peak SWE verification, the correlation coefficient between *in situ* and posterior peak snow depth is about the same at all HUC2 basins, with the highest value (*R* = 0.81) in CA. The *MD* and *RMSD* values for peak snow depth are around 2 to 3 times larger than those in peak SWE, partially caused by larger snow depth values than SWE and perhaps the poorer quality of *in situ* snow depth measurements.

### Verification with airborne snow observatory (ASO) data

The WUS–SR estimates are further verified against gridded SWE and snow depth estimates from ASO^11,56–58^. The lidar-based ASO measures snow depth via an airborne laser scanner (ALS) based on the differences in elevations between a snow-off day and snow-on days. ASO SWE is estimated from the high-resolution snow depth measurements and modeled snow density^11^. For comparison, the 50-m ASO SWE and snow depth snapshots are aggregated to the WUS–SR SWE model resolution. ASO data is available over select sites in California, Colorado, and Washington starting from 2013. While abundant snapshots are available in the Tuolumne River Basin in California, limited snapshots (commonly once per year) were taken at most of the ASO sites. ASO snow depth is a relatively accurate measurement with measurement error less than 0.02 m at a 50 m × 50 m grid. Model error (5%–8%^11^) could exist in modeled snow density, which is expected to propagate to ASO SWE estimates.

**Fig. 10:**
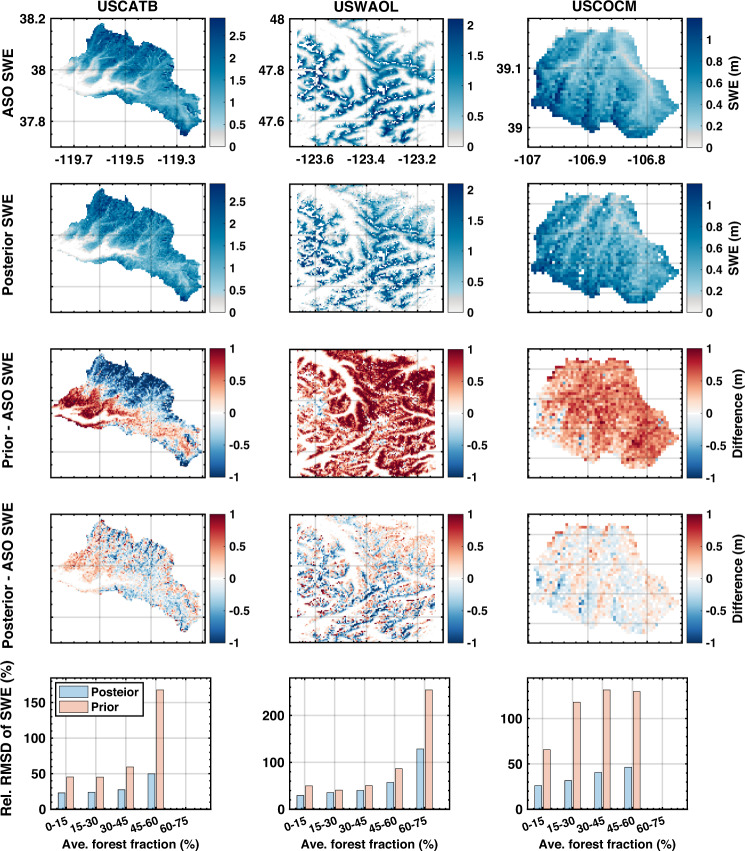
Comparison of ASO SWE with prior and posterior SWE at three ASO sites (top four rows): Tuolumne River Basin, California, (USCATB) in WY 2017 (left column); Olympic Mountains, Washington, (USWAOL) in WY 2016 (middle column); Aspen/Castle-Maroon, Colorado (USCOCM) in WY 2019 (right column). The prior maps are not shown, but instead included implicitly via the difference maps. The bottom row shows the relative *RMSD* between ASO and WUS–SR SWE as a function of forest fraction. *RMSD* (from pixels with both ASO and WUS–SR SWE greater than 1 cm) is computed for each forest fraction bin and then normalized by bin-averaged ASO SWE to get relative *RMSD*.

**Fig. 11:**
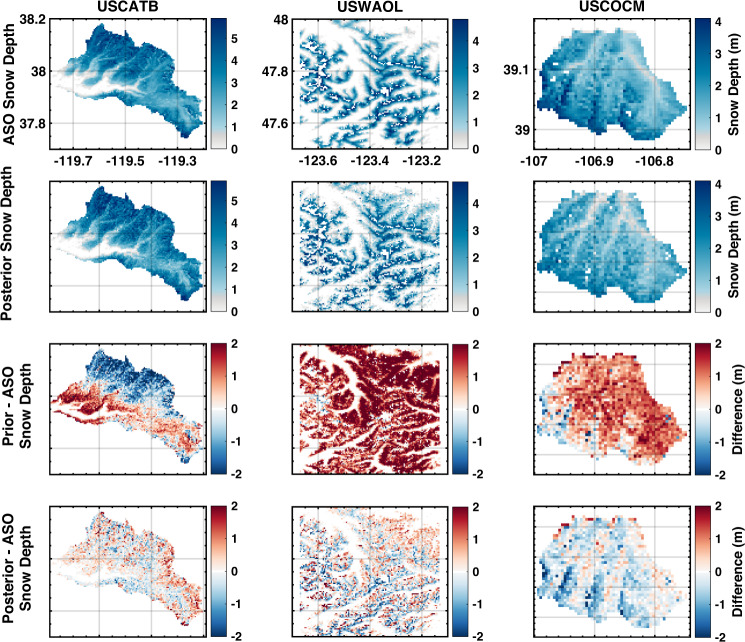
Same as Fig. 10 (top four rows) but for snow depth.

ASO SWE and snow depth estimates are compared with prior and posterior ensemble median SWE and snow depth maps on coincident days (Figs. 10 and 11). Tables 6 and 7 reports the statistical metrics for comparisons closest to April 1^st^ at sampled ASO basins: USCATB (Tuolumne River Basin, California), USWAOL (Olympic Mountains, Washington), and USCOCM (Aspen/Castle-Maroon, Colorado).

#### SWE map comparison

For the California domain (USCATB, Fig. 10 left column, Table 6), posterior SWE is highly correlated with ASO SWE (ranging from 0.81 to 0.91) compared against prior SWE (ranging from 0.50 to 0.71). A negative *MD* indicates that the WUS–SR SWE is less than ASO SWE (on average) in Tuolumne. The difference significantly decreases from prior to posterior estimates in most years, along with decreased *RMSD*. WY 2015 was a historically dry year, in which posterior SWE shows no bias compared with ASO SWE, with a small *RMSD* of 0.07 m. Posterior SWE in WY 2017 has the highest correlation (0.91) with ASO SWE compared with a lower correlation (0.56) in prior SWE. *MD* drops from −0.13 m to −0.04 m, and *RMSD* decreases by half from prior to posterior in WY 2017. Figure 10 (left column) illustrates that Tuolumne-averaged posterior SWE (1.23 m) is comparable with ASO SWE (1.27 m), suggesting that the posterior WUS–SR SWE and ASO are in good agreement with respect to the basin-wide mean SWE. The prior underestimates SWE at high elevations in the northern parts and southern edges of Tuolumne basins whereas it overestimates shallow SWE near the basin outlet. The performance of the spatially distributed posterior SWE is considerably improved over the prior compared with ASO SWE. Though *MD* in WY 2019 increases from −0.06 m to −0.14 m (from prior to posterior), *RMSD* in that year decreases from 0.34 m to 0.27 m. The differences between prior SWE and ASO SWE are large in absolute values, while large positive differences are offset by negative differences causing a low *MD* for prior SWE in WY 2019.

**Table 6:** SWE comparison statistics between ASO SWE estimates and prior and posterior (post.) snow reanalysis SWE on ASO measurement days (Day of Water Year; DOWY) closest to April 1^st^.

ASO basins	Year	DOWY	*R*		*MD* (m)		*RMSD* (m)	
			Prior	Post.	Prior	Post.	Prior	Post.
USCATB	2015	185	0.54	0.81	−0.05	0.00	0.09	0.07
	2016	184	0.59	0.83	−0.24	−0.15	0.37	0.25
	2017	183	0.56	0.91	−0.13	−0.04	0.63	0.32
	2018	205	0.63	0.82	−0.18	−0.11	0.30	0.22
	2019	175	0.62	0.84	−0.06	−0.14	0.34	0.27
	2020	196	0.71	0.88	0.03	0.05	0.14	0.13
	2021	211	0.50	0.82	−0.12	0.03	0.18	0.13
USWAOL	2016	181	0.81	0.81	0.53	−0.03	0.68	0.38
USCOCM	2019	189	0.45	0.75	0.41	0.01	0.47	0.17

USCATB represents the Tuolumne River Basin (California); USWAOL represents the Olympic Mountains (Washington); USCOCM represents Aspen/Castle-Maroon (Colorado).

Non-seasonal SWE in portions of the PN (USWAOL) site is a potential error source in both ASO SWE and WUS–SR SWE. Snow depth retrieved from ASO may be erroneous at glacier pixels due to the lack of snow-off flights. The snow reanalysis framework does not include explicit modeling of glaciers. Therefore, non-seasonal snow pixels are removed when comparing the ASO SWE with WUS–SR SWE. This paper generates the WUS–SR non-seasonal snow mask following the method described in Liu *et al*.^50^. To summarize the method herein, a pixel is considered as a non-seasonal snow pixel if the annual minimum SWE exceeds 10% of the annual maximum SWE at least once over the dataset period. After applying the non-seasonal snow mask, the mean posterior SWE is 0.51 m which is slightly lower than 0.55 m in ASO SWE. Though the correlation coefficient is high (over 0.8) between prior snow reanalysis SWE and ASO SWE, the *MD* and *RMSD* in absolute value is over 0.50 m and 0.60 m respectively, which are both reduced significantly (by 94% and 44% respectively) in the posterior.

In Colorado (USCOCM), the mean of posterior SWE (0.55 m) is comparable with ASO SWE (0.54 m). The *MD* is reduced by 98% (to 0.01 m) and *RMSD* is reduced by 64% (to 0.17 m) from prior to posterior estimates. Although the posterior correlation coefficient is significantly improved over the prior, it is lower than the values seen at the USCATB and USWAOL sites. In Colorado, snow albedo has been shown to be affected by dust, black carbon, and other light-absorbing particles in recent decades^59^. In the current snow reanalysis framework, the impact of dust on snow albedo is modeled through an unconstrained uncertainty parameter. Future work could be done to apply a more explicit treatment of dust impacts on snow albedo to yield potentially improved results.

The effect of forest fraction on the performance of reanalysis SWE estimates is further illustrated using ASO SWE in Fig. 10. The Olympics basin has denser forest fraction with a mean of 58%, while the Tuolumne and Aspen/Castle-Maroon basins have mean forest fractions of 17% and 20%, respectively. At all three ASO basins, the relative RMSD of posterior SWE increases with the forest fraction. This is expected since Landsat-derived fSCA is only available over bare areas and/or forest gaps within a pixel. As forest cover increases, less useful information is available, while information is maximized at 0% of forest cover. However, the improvement in prior to posterior SWE estimates increases with forest coverage. This is likely related to the increased complexity of modeling SWE in dense forest areas where the larger uncertainty in forest areas is still reduced with the assimilation of fSCA.

#### Snow depth map comparison

Similar to the SWE comparison, posterior snow depth is verified against the ASO snow depth measurements (Fig. 11, Table 7). The spatial distribution of snow depth differences is comparable to the SWE differences with a correlation coefficient (*R*) of 0.85 and 0.76 in Washington and Colorado, respectively, and a value above 0.82 in California. In California, the *MD* of posterior snow depth is reduced by over 30% and *RMSD* is reduced by over 20% compared to the statistics of prior snow depth over WY 2015 to 2018, and WY 2021. In WY 2019 and 2020, while the posterior *MD* values are larger than the prior *MD* (positive and negative differences cancel each other out), the *R* values are as high as 0.9, and the *RMSD* values are reduced by 28% and 30%, respectively. In Washington, the posterior *MD* is close to 0 with *RMSD* significantly reduced by over 50% from the prior to the posterior estimates. In Colorado, despite the absolute values of *MD* and *RMSD* for posterior snow depth being more than twice the values of posterior SWE statistics (due to the larger dynamic range), the estimation of posterior snow depth is significantly improved from the prior snow depth with *MD* and *RMSD* reduced by 60% and 40%, respectively.

**Table 7:** Same as Table 6 but for snow depth.

ASO basins	Year	DOWY	*R*		*MD* (m)		*RMSD* (m)	
			Prior	Post.	Prior	Post.	Prior	Post.
**USCATB**	2015	185	0.54	0.82	−0.10	−0.01	0.21	0.14
	2016	184	0.73	0.90	−0.38	−0.18	0.69	0.41
	2017	183	0.61	0.92	−0.13	0.09	1.16	0.62
	2018	205	0.71	0.83	−0.32	−0.18	0.60	0.46
	2019	175	0.72	0.90	−0.01	−0.20	0.78	0.56
	2020	196	0.75	0.90	0.02	0.05	0.43	0.30
	2021	211	0.50	0.82	−0.33	0.04	0.47	0.29
**USWAOL**	2016	181	0.81	0.85	1.34	0.02	1.64	0.76
**USCOCM**	2019	189	0.54	0.76	0.75	−0.30	1.03	0.62

## Usage Notes

The snow reanalysis framework described herein is designed to capture seasonal snow in mountainous areas and does not model glacier processes. However, some non-seasonal snow and glaciers may exist in some regions of the WUS (e.g., the Olympics). Such grid cells can be diagnosed and excluded as described above using the non-seasonal snow mask (as diagnosed via the snow reanalysis framework). It is recommended to mask out these pixels before comparing with other datasets for seasonal snow.

The WUS–SR dataset is developed from a LSM-SDC model integrated with fSCA data assimilation. The strength of the product is in its space-time continuity where highly uncertain model-based prior estimates are constrained by snow measurements. While uncertainty and bias correction are embedded in the framework, errors and uncertainty in MERRA2 forcings, model parameters, and Landsat fSCA retrievals undoubtedly affect the accuracy of SWE estimates. Developing the uncertainty models using sparse *in situ* data and application of uncertainty model parameters uniformly over space, likely both oversimplify the true uncertainty and how it varies across different physiographic and climatological gradients.

Compared with the previous framework and inputs used in the published 90-m reanalysis dataset over the Sierra Nevada^33^, some key updates/changes in the current snow reanalysis framework include: (1) use of MERRA2 forcings instead of NLDAS2 forcings, which are globally available and were found to yield marginally better SWE estimates relative to ASO estimates in Tuolumne^34^; (2) use of the SRTM DEM (with the ASTER DEM used for void filling) and (3) use of the globally available AVHRR landcover map instead of the National Land Cover Database limited in the U.S. These changes are primarily made to use globally available data for extension and application to broad spatial domains.

Future versions of this dataset could include: (1) use of multi-source fSCA measurements from Landsat, MODIS, Sentinel, and/or other sources (e.g., VIIRS) to increase the number of cloud-free fSCA images (especially in the Pacific Northwest); (2) examination of the impact of different forcings (e.g., NLDAS2, MERRA2, ERA5) and their uncertainties; (3) use of time-varying forest cover to better reflect transient changes; (4) use of dust-on-snow measurements^60^ to better constrain albedo; and (5) use of multi-resolution approaches^61,62^ to better capture snow estimates in complex terrain with higher resolution where necessary; (6) use of fSCA from Landsat 9 in the future versions.

## Data Availability

To aid in usage of the dataset, code to generate sample plots and verification figures contained herein are publicly available on GitHub (https://github.com/yiwenff/WUS-SR-data-descriptor).

## References

[1] Sturm M, Goldstein MA, Parr C (2017). Water and life from snow: A trillion dollar science question. Water Resour. Res..

[2] Li D, Wrzesien ML, Durand M, Adam J, Lettenmaier DP (2017). How much runoff originates as snow in the western United States, and how will that change in the future?. Geophys. Res. Lett..

[3] Huning LS, AghaKouchak A (2020). Global snow drought hot spots and characteristics. Proc. Natl. Acad. Sci..

[4] Yan H (2020). Evaluating next-generation intensity–duration–frequency curves for design flood estimates in the snow-dominated western United States. Hydrol. Process..

[5] Hamilton AL, Characklis GW, Reed PM (2020). Managing Financial Risk Trade-Offs for Hydropower Generation Using Snowpack-Based Index Contracts. Water Resour. Res..

[6] Larson KM (2009). Can we measure snow depth with GPS receivers?. Geophys. Res. Lett..

[7] Nolin, A. W. *et al*. New snow metrics for a warming world. *Hydrol. Process*. **35** (2021).

[8] Molotch NP, Bales RC (2006). SNOTEL representativeness in the Rio Grande headwaters on the basis of physiographics and remotely sensed snow cover persistence. Hydrol. Process..

[9] Andreadis KM, Lettenmaier DP (2006). Assimilating remotely sensed snow observations into a macroscale hydrology model. Adv. Water Resour..

[10] Markus T (2017). The Ice, Cloud, and land Elevation Satellite-2 (ICESat-2): Science requirements, concept, and implementation. Remote Sens. Environ..

[11] Painter TH (2016). The Airborne Snow Observatory: Fusion of scanning lidar, imaging spectrometer, and physically-based modeling for mapping snow water equivalent and snow albedo. Remote Sens. Environ..

[12] Lievens H (2019). Snow depth variability in the Northern Hemisphere mountains observed from space. Nat. Commun..

[13] Yueh, S. *et al*. UAS-based P-band signals of opportunity for remote sensing of snow and root zone soil moisture. in *Sensors, Systems, and Next-Generation Satellites XXII* vol. 10785 107850B (International Society for Optics and Photonics, 2018).

[14] Shi J, Dozier J (2000). Estimation of snow water equivalence using SIR-C/X-SAR. II. Inferring snow depth and particle size. IEEE Trans. Geosci. Remote Sens..

[15] Nghiem SV, Tsai W-Y (2001). Global snow cover monitoring with spaceborne K/sub u/-band scatterometer. IEEE Trans. Geosci. Remote Sens..

[16] Hersbach H (2020). The ERA5 global reanalysis. Q. J. R. Meteorol. Soc..

[17] Muñoz-Sabater J (2021). ERA5-Land: a state-of-the-art global reanalysis dataset for land applications. Earth Syst. Sci. Data.

[18] Kobayashi S (2015). The JRA-55 Reanalysis: General Specifications and Basic Characteristics. J. Meteorol. Soc. Jpn. Ser II.

[19] Rodell M (2004). The Global Land Data Assimilation System. Bull. Am. Meteorol. Soc..

[20] Gelaro R (2017). The Modern-Era Retrospective Analysis for Research and Applications, Version 2 (MERRA-2). J. Clim..

[21] Luojus K (2021). GlobSnow v3.0 Northern Hemisphere snow water equivalent dataset. Sci. Data.

[22] Wrzesien ML, Pavelsky TM, Durand MT, Dozier J, Lundquist JD (2019). Characterizing Biases in Mountain Snow Accumulation From Global Data Sets. Water Resour. Res..

[23] Xu Y, Jones A, Rhoades A (2019). A quantitative method to decompose SWE differences between regional climate models and reanalysis datasets. Sci. Rep..

[24] Kim RS (2021). Snow Ensemble Uncertainty Project (SEUP): quantification of snow water equivalent uncertainty across North America via ensemble land surface modeling. The Cryosphere.

[25] National Operational Hydrologic Remote Sensing Center. *Snow Data Assimilation System (SNODAS) Data Products at NSIDC, Version 1*. (2004).

[26] Zeng X, Broxton P, Dawson N (2018). Snowpack Change From 1982 to 2016 Over Conterminous United States. Geophys. Res. Lett..

[27] Fang Y, Liu Y, Margulis S (2022). NASA Earth Data.

[28] Huning LS, AghaKouchak A (2020). Approaching 80 years of snow water equivalent information by merging different data streams. Sci. Data.

[29] Margulis SA (2016). Characterizing the extreme 2015 snowpack deficit in the Sierra Nevada (USA) and the implications for drought recovery. Geophys. Res. Lett..

[30] Li D, Lettenmaier DP, Margulis SA, Andreadis K (2019). The Value of Accurate High-Resolution and Spatially Continuous Snow Information to Streamflow Forecasts. J. Hydrometeorol..

[31] Pflug JM, Margulis SA, Lundquist JD (2022). Inferring watershed-scale mean snowfall magnitude and distribution using multidecadal snow reanalysis patterns and snow pillow observations. Hydrol. Process..

[32] Margulis SA, Girotto M, Cortés G, Durand M (2015). A Particle Batch Smoother Approach to Snow Water Equivalent Estimation. J. Hydrometeorol..

[33] Margulis SA, Cortés G, Girotto M, Durand M (2016). A Landsat-Era Sierra Nevada Snow Reanalysis (1985–2015). J. Hydrometeorol..

[34] Margulis SA, Liu Y, Baldo E (2019). A Joint Landsat- and MODIS-Based Reanalysis Approach for Midlatitude Montane Seasonal Snow Characterization. Front. Earth Sci..

[35] Sun S, Xue Y (2001). Implementing a new snow scheme in Simplified Simple Biosphere Model. Adv. Atmospheric Sci..

[36] Xue, Y., Sun, S., Kahan, D. S. & Jiao, Y. Impact of parameterizations in snow physics and interface processes on the simulation of snow cover and runoff at several cold region sites. *J. Geophys. Res. Atmospheres* **108** (2003).

[37] Sun S, Jin J, Xue Y (1999). A simplified layer snow model for global and regional studies. J Geophys Res.

[38] Dickinson RE, Henderson-Sellers A, Kennedy PJ (1993). Biosphere-Atmosphere Transfer Scheme (BATS) version 1e as coupled to the NCAR community climate model. Technical note. [NCAR (National Center for Atmospheric Research)]..

[39] Liston GE (2004). Representing Subgrid Snow Cover Heterogeneities in Regional and Global Models. J. Clim..

[40] Farr, T. G. *et al*. The Shuttle Radar Topography Mission. *Rev. Geophys*. **45** (2007).

[41] NASA. *Shuttle Radar Topography Mission (SRTM)*. https://www2.jpl.nasa.gov/srtm/ (2013).

[42] NASA. *ASTER*. http://asterweb.jpl.nasa.gov/ (2001).

[43] Hansen MC, Defries RS, Townshend JRG, Sohlberg R (2000). Global land cover classification at 1 km spatial resolution using a classification tree approach. Int. J. Remote Sens..

[44] (2017). U.S. Geological Survey.

[45] Sexton JO (2013). Global, 30-m resolution continuous fields of tree cover: Landsat-based rescaling of MODIS vegetation continuous fields with lidar-based estimates of error. Int. J. Digit. Earth.

[46] Sexton, J. O. *et al*. *Global 30m Landsat Tree Canopy Version 4*. https://e4ftl01.cr.usgs.gov/MEASURES/GFCC30TC.003/.

[47] Painter TH, Dozier J, Roberts DA, Davis RE, Green RO (2003). Retrieval of subpixel snow-covered area and grain size from imaging spectrometer data. Remote Sens. Environ..

[48] Cortés G, Girotto M, Margulis SA (2014). Analysis of sub-pixel snow and ice extent over the extratropical Andes using spectral unmixing of historical Landsat imagery. Remote Sens. Environ..

[49] USGS. *SLC-off Gap-Filled Products Gap-Fill Algorithm Methodology*. https://www.usgs.gov/media/files/landsat-7-slc-gap-filled-products-phase-two-methodology (2004).

[50] Liu Y, Fang Y, Margulis SA (2021). Spatiotemporal distribution of seasonal snow water equivalent in High Mountain Asia from an 18-year Landsat–MODIS era snow reanalysis dataset. The Cryosphere.

[51] Margulis SA, Fang Y, Li D, Lettenmaier DP, Andreadis K (2019). The Utility of Infrequent Snow Depth Images for Deriving Continuous Space-Time Estimates of Seasonal Snow Water Equivalent. Geophys. Res. Lett..

[52] Cortés G, Margulis S (2017). Impacts of El Niño and La Niña on interannual snow accumulation in the Andes: Results from a high-resolution 31 year reanalysis. Geophys. Res. Lett..

[53] Girotto M, Margulis SA, Durand M (2014). Probabilistic SWE reanalysis as a generalization of deterministic SWE reconstruction techniques: PROBABILISTIC SWE REANALYSIS. Hydrol. Process..

[54] Liu Y, Margulis SA (2019). Deriving Bias and Uncertainty in MERRA-2 Snowfall Precipitation Over High Mountain Asia. Front. Earth Sci..

[55] Girotto M, Cortés G, Margulis SA, Durand M (2014). Examining spatial and temporal variability in snow water equivalent using a 27 year reanalysis: Kern River watershed, Sierra Nevada. Water Resour. Res..

[56] Painter T (2018). NASA National Snow and Ice Data Center DAAC.

[57] Painter T (2018). NASA National Snow and Ice Data Center DAAC.

[58] *Airborne Snow Observatories, Inc*. https://data.airbornesnowobservatories.com/ (2020).

[59] Deems JS, Painter TH, Barsugli JJ, Belnap J, Udall B (2013). Combined impacts of current and future dust deposition and regional warming on Colorado River Basin snow dynamics and hydrology. Hydrol. Earth Syst. Sci..

[60] Skiles SM (2015). Regional variability in dust-on-snow processes and impacts in the Upper Colorado River Basin. Hydrol. Process..

[61] Baldo E, Margulis SA (2018). Assessment of a multiresolution snow reanalysis framework: a multidecadal reanalysis case over the upper Yampa River basin, Colorado. Hydrol. Earth Syst. Sci..

[62] Baldo E, Margulis SA (2017). Implementation of a physiographic complexity-based multiresolution snow modeling scheme: MULTIRESOLUTION SNOW MODELING. Water Resour. Res..

[63] Global Modeling and Assimilation Office (GMAO). *MERRA-2, version 5.12.4*., 10.5067/VJAFPLI1CSIV (2015).

[64] USGS. *Landsat*. http://earthexplorer.usgs.gov (1984).

